# The metabolism of amino acids, AsA and abscisic acid induced by strigolactone participates in chilling tolerance in postharvest zucchini fruit

**DOI:** 10.3389/fpls.2024.1402521

**Published:** 2024-05-14

**Authors:** Lei Wang, Li Liu, Anqi Huang, Hua Zhang, Yonghua Zheng

**Affiliations:** ^1^ College of Agriculture and Agricultural Engineering, Liaocheng University, Liaocheng, China; ^2^ College of Food Science and Technology, Nanjing Agricultural University, Nanjing, China

**Keywords:** *Cucurbita pepo*, chilling tolerance injury, strigolactone, ABA, physiological metabolism

## Abstract

Zucchini fruit are notably susceptible to chilling injury when stored at low temperatures. The purpose of this experimental investigation was to assess the influence of strigolactone (ST) (5 μmol L^-1^) on mitigating chilling injury and the metabolic changes in amino acids, ascorbic acid, and abscisic acid in zucchini fruit stored at 4°C. Research findings demonstrated that ST-treated zucchini fruit displayed a significantly higher tolerance to chilling stress compared to the control group. Postharvest ST treatment led to a decrease in weight loss, accompanied by reduced levels of malondialdehyde and relative ion leakage compared to the untreated group. ST immersion significantly boosted the metabolic pathways associated with proline and arginine, affecting both the enzymatic reactions and gene expressions, thus cumulatively increasing the internal concentrations of these amino acids in zucchini fruit. Zucchini treated with ST exhibited an increased concentration of γ-aminobutyric acid (GABA) as a result of augmented activities and elevated transcriptional levels of glutamate decarboxylase (GAD), GABA transaminase (GAT), and succinate semialdehyde dehydrogenase (SSD). In the ST-treated sample, the elevated enzymatic activities and enhanced gene expressions within the ascorbic acid (AsA) biosynthesis pathway worked together to sustain AsA accumulation. The application of ST resulted in a rise in abscisic acid (ABA) concentration, which correspondingly correlated with the induction of both activities and gene expression levels of crucial enzymes involved in ABA metabolism. Our findings revealed that submerging zucchini fruit in ST could be a highly effective strategy for boosting their chilling tolerance. The alleviation in chilling injury induced by ST may be attributed to the modulation of proline, arginine, GABA, AsA and ABA metabolism.

## 1 Introduction

Among the multitude of postharvest preservation techniques for fruit and vegetable, cold storage continues to be recognized as the most efficacious method and is extensively employed for the storage and conservation of horticultural commodities (Valenzuela et al., 2017; Zhang et al., 2019). Cold storage effectively mitigates the metabolic activity in plant cells, postpones senescence, and safeguards the quality of postharvest horticultural goods. Nonetheless, this method poses a challenge for certain fruit and vegetable native to tropical and subtropical regions, as it can induce chilling injury (CI), thereby resulting in a decline in their overall quality (Zhang et al., 2019). Zucchini (*Cucurbita pepo* L.) is a highly nutritious and medicinally valuable vegetable crop that is notably sensitive to chilling temperatures (Palma et al., 2019; Zuo et al., 2021). The manifestations of CI in zucchini fruit are predominantly characterized by the emergence of pits and damaged patches on the outer surface or exocarp tissue (Carvajal et al., 2015). The appearance of these CI symptoms causes zucchini to be very susceptible to disease infection and rot, which directly affects its commercial value. Consequently, exploring methods to prevent or alleviate CI in zucchini fruit has emerged as a pressing research focus within the field of postharvest science. Experimental studies have discovered that specific postharvest treatments are capable of effectively reducing the severity of chilling injury in postharvest fruit and vegetable (Aghdam et al., 2019; Islam et al., 2022). Previous studies have proposed that the potential biochemical mechanism of chilling injury in fruit and vegetable involve several interconnected processes: disruption to cellular membranes, reactive oxygen species (ROS) stress, alterations in unsaturated fatty acid metabolism, perturbations in energy supply, and changes in intracellular sugar metabolism (Wu et al., 2024). Current research also attempts to elucidate the mechanism of CI in terms of the role of internal signaling molecules and functional phytochemicals within fruit and vegetable, such as GABA, proline, and argnine.

Plants inherently hold the potential capacity to cope with adverse environmental situations. Exogenous stimuli can efficaciously activate this complex adaptation process, which unfolds through the meticulous choreography of a wide array of internal signaling molecules. Strigolactone (ST), a category of phytohormones derived from carotenoids, serve as vital small signaling molecules governing various developmental stages and responding to a multitude of environmental cues (Soliman et al., 2022). Beyond their established functions in modulating plant growth and nutrient distribution within plants, ST’ positive influence on the postharvest physiological processes of fruits has garnered escalating interest in their scientific investigation (Wu et al., 2022; Liu et al., 2024a). During refrigerated storage at 0°C, ST enhanced fruit quality in strawberries by bolstering the antioxidant defense system and regulating the metabolic pathways involving phenylpropanoids, nitric oxide, and hydrogen sulfide (Huang et al., 2021a). Physiological evaluations demonstrated that the use of ST significantly alleviated the progression of CI in postharvest litchi fruit (Liu et al., 2024a). Ferrero et al. (2018) highlighted the involvement of ST in enhancing the accumulation of anthocyanins within grapevine berries through synergistic interactions with other phytohormones. It is becoming increasingly clear that ST is finding broader applications in agriculture contexts (Li et al., 2023; Kapoor et al., 2024). Considering the extensive applications of ST in agricultural practices, obtaining a thorough comprehension of its multifaceted roles across a broad range of plant species is essential. Up until now, no scientific research has been published detailing the effects of ST immersion treatments in reducing chilling damage during the postharvest period for zucchini fruit. This study aimed to explore the efficacy of ST in diminishing chilling injury in zucchini fruit during cold storage. Concurrently, we scrutinized the metabolic changes in amino acids, ascorbic acid and abscisic acid to shed light on the potential biochemical mechanisms responsible for the improved chilling tolerance in postharvest zucchini fruit during cold storage treated with ST.

## 2 Materials and methods

### 2.1 *ST treatment and* sample collection

Fruit (*Cucurbita pepo* L. cv. Zaoqing) were meticulously handpicked from a farm situated in the outskirts of Liaocheng at their commercial maturity stage. Any fruit found to have defects were carefully discarded to ensure only top-quality produce was used. Subsequently, we selected fruit that displayed consistent size and uniform coloration. All zucchini fruit were divided randomly into two sets of 150 pieces each. Based on a preliminary screening test conducted over a range of concentrations (0, 1, 5, 10, 20 μM), one set was treated by immersing them in a solution consisting of 5 μM ST (Solarbio Science and Technology Co., Ltd., Beijing, China) for a period of ten minutes. This experimental process validated that the chosen concentration of 5 μM ST exerted the most efficacious results. Another batch was treated with sterile water instead, functioning as the control group. Thereafter, all the fruit were left to dry out gently at the ambient temperature of 20°C for around an hour. The fruit were then stored at 4°C for a span of sixteen days, under conditions where the relative humidity was consistently held at 90%. A total of 30 fruit from each batch were shifted to 20°C for a 2-day shelf life simulation before undergoing physio-biochemical index assessments and chilling injury measurements every four days. For each replicate, the sample tissue of ten fruit without chilling injury symptom was mixed together and stored in liquid nitrogen for subsequent physio-biochemical parameter analysis. Each treatment had three biological replicates at every assessment point, and the entire experiment was repeated twice.

### 2.2 Assessment of chilling injury in cold-stored zucchini

The severity of chilling injury was determined by evaluating the external surface pitting areas in zucchini fruit. The classification system for assessing the severity of chilling injury was structured as follows: At Grade 0, there were no discernible symptoms of pitting. Grade 1 denoted that the affected area covered less than 10% of the fruit’s total surface. In Grade 2, the pitting extended across 11% to 25% of the fruit’s skin. Grade 3 represented a pitting area ranging from 26% to 50% of the fruit’s surface. Lastly, Grade 4 signified that the pitting had spread over more than 50% of the fruit’s exterior. The Chilling Injury Index was calculated using the following formula: ∑(chilling injury level × quantity of fruit at corresponding level)/(4 × total fruit number).

### 2.3 Quantifications of malondialdehyde (MDA) and relative and ion leakage

To assess the MDA content and ion leakage in the experimental fruit, we adhered to the detailed protocol outlined by Wang et al. (2023). A sample of fruit pulp was ground in a 15 mL of TCA solution. The resultant mixture was then centrifuged at 10,000 g for a duration of 15 minutes. Following this, the absorbance of the extracted supernatant was measured spectrophotometrically at three distinct wavelengths, namely 450, 532, and 600 nm. The MDA concentration was computed using the following formula: 6.45×(Ab_532_-Ab_600_)-0.56×Ab_450_. To determine ion leakage, four grams of uniformly sliced zucchini cylinders (with a thickness of 2 mm) taken from the cuticular layer of ten fruits were soaked in 30 mL of deionized water for a period of ten minutes. The initial electrical conductivity (D_i_) was then recorded. Next, the samples were boiled for another ten minutes before cooling down to 20°C. Upon reaching this temperature, the final electroconductivity (D_f_) was measured in another 30 mL of deionized water. The ion leakage percentage was derived from the ratio of initial to final conductivity values, expressed as: (D_i_/D_f_) × 100%.

### 2.4 Measurement of proline content and the activities of enzymes involved in proline metabolism

The determination of proline content was carried out according to the previous research of Wang et al. (2023). Tissue sample was homogenized in 20 mL of sulphosalicylic acid and then subjected to water bath incubation at 100°C for tenminutes. Following a 10-minute centrifugation at 15,000 g, the supernatant was retrieved and mixed with methylbenzene (5 mM), ninhydrin (2%, v/v), and acetic acid (5%, v/v). The absorbance of the reaction mixture was measured at 520 nm by spectrophotometry, with results presented in terms of mg/kg of tissue sample.

Ornithine-δ-aminotransferase (OAT) activity was measured following the procedure outlined in the research of Wang et al. (2023). Tissue sample was homogenized in tripotassium phosphate buffer containing dithiothreitol, and the mixture was subsequently centrifuged at 15,000 g for 12 minutes at 4°C. The assay mixture was composed of crude enzyme (0.8 mL), 1.3 mL of Orn (35 mM), 0.2 mL of ninhydrin (2%, w/v), and 1.7 mL of phosphopyridoxal (0.15 mM). One unit of enzyme activity is defined as an increment of 0.01 absorbance units at 510 nm per minute, which is recorded in U/kg tissue sample.

Tissue sample was crushed in Tris-HCl buffer solution including PVP, EDTA, and dithiothreitol, which was then centrifuged at 15,000 g for 15 minutes at 4°C. Reaction mixture for measuring PCS (pyrroline-5-carboxylate synthase) consisted of enzyme supernatant, MgCl_2_, Tris-HCl buffer, and ATP. Reaction mixture for measuring PDH (proline dehydrogenase) consisted of enzyme supernatant, proline, Na_2_CO_3_-NaHCO_3_ buffer, and NAD^+^ (Wang et al., 2023). One unit of activity of the above two enzymes is defined as the change in absorbance by 0.001 per minute at a wavelength of 340 nm.

### 2.5 Measurements of GABA content and the activities of enzymes involved in GABA metabolism

GABA concentration was determined following the analytical methods outlined by Madebo et al. (2021). Zucchini tissue was homogenized in a 50 mM lanthanum chloride solution, and the mixture was subsequently centrifuged at 15,000 g for 12 minutes at 4°C. The assay blend included 0.6 mL boracic acid buffer (pH 9.0), 0.8 mL phenyl hydroxide, 0.6 mL NaClO_4_, 0.4 mL potassium hydroxide, and 2.6 mL obtained supernatant. The absorbance of the assay blend was measured spectrophotometrically at a wavelength of 645 nm, with results presented in terms of milligrams per kilogram of tissue sample.

Glutamate decarboxylase (GAD) activity was measured following the procedure outlined in the research of Madebo et al. (2021). Zucchini tissue was crushed in Tris-HCl buffer solution, which was then centrifuged at 15,000 g for 15 minutes at 4°C. The assay mixture consisted of tripotassium phosphate buffer (0.25 M), glycerol (0.1 mM), phosphopyridoxal (0.07 mM), dithiothreitol (0.1 mM). One unit of enzyme activity is defined as the production of one microgram of GABA within one minute. GABA transaminase (GAT) activity was measured following the procedure outlined in the research of Madebo et al. (2021). Zucchini tissue was crushed in PBS buffer, which was then centrifuged at 15,000 g for 15 minutes at 4°C. The assay mixture consisted of GABA (0.1 mM), DTT (0.2 mM), ethylene diamine tetraacetic acid (0.03 M), acetonate (0.05 mM), phosphopyridoxal (0.05 mM), Tris-HCI buffer (0.2 M), obtained supernatant (0.6 mL). The reaction was terminated by adding sulfosalicylic acid, and the absorbance of the solution was subsequently determined spectrophotometrically at a wavelength of 450 nm. The determination of succinate semialdehyde dehydrogenase (SSD) activity was conducted in adherence to the guidelines provided in the Plant SSD ELISA Kit from Jiangsu Boshen Biotechnology Co., LTD. Tissue sample was crushed in PBS buffer (pH 7.3), which was then centrifuged at 15,000 g for 15 minutes at 4°C. The assay mixture consisted of POD, DTT, tetramethylbenzidine, and obtained supernatant. The absorbance of the mixture was measured spectrophotometrically at a wavelength of 450 nm, with results presented in terms of units per gram of tissue sample.

### 2.6 Measurements of AsA content and the activities of enzymes involved in AsA metabolism

The determination of AsA concentration in zucchini fruit was guided by the methods outlined in the work of Wang et al. (2016). To measure the AsA concentration, four grams of fruit tissues were finely ground in 15 milliliters of prechilled oxalic acid. Subsequently, the AsA content was determined through a titration process using 2,6-dichlorophenol indophenol as the titrating agent. The result was reported in units of grams per kilogram of tissue. The measurement of ascorbate peroxidase (APX) activity was conducted according to the protocol laid out by Li et al. (2019). The composition of the reaction blend included enzyme suspension, sodium phosphate buffer (1.5 mL, 50 mM, pH 7.0), AsA (50 µL, 9 mM), and H_2_O_2_ (15 µL, 30%). The unit defining enzyme activity was considered as the amount of enzyme that caused a decrement in absorbance at a wavelength of 290 nm min^-1^. The quantification of monodehydroascorbate reductase (MDR) activity was performed according to the methodologies prescribed by Liu et al. (2024b). Zucchini tissue was homogenized in prechilled Tris-HCl buffer that included polyvinylpyrrolidone (5%, w/v), ethylenediaminetetraacetic acid, Triton X-100, and dithiothreitol. Following this, the mixture was subjected to centrifugation at 11,000 g (4°C). The composition of the reaction mixture included HEPES-KOH buffered solution, NADH, ascorbate, ascorbate oxidase enzyme, and 0.8 milliliters of the supernatant derived from the enzyme preparation. The result was determined by measuring absorbance at a wavelength of 340 nm using a spectrophotometer. The assessment of ascorbate oxidase (ACO) activity in the fruit tissue was conducted in accordance with the methodology prescribed by Yun et al. (2020). Fruit pulp were finely ground in a pre-cooled sodium phosphate buffer solution (pH 6.5). The reaction blend primarily comprised ascorbic acid, glutathione, and the supernatant fraction. The composition of the reaction mixture included glutathione, AsA, and supernatant derived from the enzyme preparation. One unit of ACO activity was established as the measure of the oxidative transformation of ascorbic acid occurring per second, and the results were expressed in terms of μmol s^-1^ kg^-1^ protein.

### 2.7 Measurements of arginine content and the activities of enzymes involved in arginine metabolism

The determination of arginine content within zucchini tissue was estimated following the method of Jin et al. (2019). Tissue sample was pulverized with hydrochloric acid solution (0.2 M), and the mixture was subsequently centrifuged at 8,000 g for 12 minutes at 4°C to collect the supernate. The residue was extensively rinsed with hydrochloric acid solution (0.2 M) and subsequently subjected to centrifugation at 8,000 g. The resulting supernatant was combined and mixed with 5-sulfosalicylic acid. After undergoing ultrasonic treatment and subsequent centrifugation at 8,000 g, the reaction system was meticulously filtered using a 0.22 μM aqueous phase filter. The filter liquor was detected with a Hitachi L-8800 amino acid analyzer and the concentration of arginine was quantified following the manufacturer’s standard procedures. Arginase (ARG) activity was measured following the procedure outlined in the research of Zhang et al. (2013). Tissue sample was crushed in Tris-HCl buffer solution including ME (2-mercaptoethanol), PMSF, and PVP, which was then centrifuged at 15,000 g for 15 min at 4°C. Reaction solution included crude enzyme, arginine, Gly-NaOH, and manganese chloride. The reaction was ceased by the addition of HClO_4_. The resulting solution was combined with another liquid containing both sulfuric and phosphoric acids, and then the mixture was incubated in boiling water bath for 60 minutes. The absorbance of reaction mixture was recorded at 520 nm. One unit of ARG activity was defined as the production of one milligram of urea per minute per gram of tissue sample. The activities of arginine decarboxylase (ADC) and ornithine decarboxylase (ODC) were performed following the protocol provided by Zhang et al. (2013). Tissue sample was crushed in sodium phosphate buffer solution including DTT, PMSF, EDTA, and AsA, which was then centrifuged at 15,000 g for 15 min at 4°C. Reaction solution included crude enzyme, PLP, EDTA, and DTT. The assay reactions were commenced by introducing 100 μL of arginine for ADC activity measurement or L-ornithine for ODC activity assessment, and the resulting mixtures were then maintained at 37°C for 30 minutes. Termination of the reaction was achieved by adding perchloric acid, following which the mixture was centrifuged at 10,000 g to collect the supernate. The absorbance of assay blend was recorded at 254 nm. Enzyme activities were represented by the variation of 0.01 units in absorbance per minute for every gram of tissue sample.

### 2.8 Measurements of ABA content and the activities of enzymes involved in ABA metabolism

The evaluation of ABA content in the frozen zucchini tissues was conducted in accordance with the standards delineated by Siebeneichler et al. (2022). To do this, 2.0 grams of zucchini tissues were thoroughly pulverized in a pre-chilled 80% methanol solution. The resulting mixtures were centrifugated at 13,000 g for 30 minutes (4°C). The resulting supernatant was passed through a 0.22-micron syringe filter for purification. After eliminating polar constituents using a Sep-Pak C18 cartridge, ABA content was measured using the enzyme-linked immunosorbent assay (ELISA) kits provided by Adanti Biotechnology Co., Ltd., Hubei, China, and expressed as µg kg^-1^ sample. Abscisic acid aldehyde oxidase (AAO) activity was determined as directed by Zuo et al. (2021). In this process, 2.5 grams of fruit specimen were ground in 10 mL prechilled phosphoric acid buffer. The resulting mixtures were subjected to centrifugation at 12,000 g for a duration of fifteen minutes at 4°C. The AAO activity was assayed spectrophotometrically at 450 nm following the producer’s instructions for AAO ELISA technique; with one unit of AAO activity defined as the generation of one nmol of ABA under the given assay conditions. The 9-cis-epoxycarotenoid dioxygenase (ECD) activity was assessed following the direction of Huang et al. (2021c). A 2.5 gram portion of fruit tissue was crushed in 15 mL prechilled PBS buffer solution and centrifuged at 12,000 g at 4°C. ECD activity was subsequently quantified spectrophotometrically at 450 nm, adhering to the instructions provided by the ELISA kit from Mlbio, Shanghai, China.

### 2.9 RNA extraction and quantitative analysis of genes expressions

RNA extraction from zucchini tissues was conducted following the methodology described by Liu et al. (2024b). First, 5.0 grams of fruit sample were ground into powder in liquid nitrogen. Total RNA was retrieved by employing a CTAB-based extraction solution containing 2% (v/v) β-mercaptoethanol. The quantity and quality of the isolated RNA were assessed utilizing a Thermo NanoDrop 2000 spectrophotometer. Subsequently, cDNA synthesis was performed through reverse transcription in adherence to the protocol recommended by Yeasen Biotechnology Co., Ltd., Shanghai. Real-time quantitative PCR (qPCR) was carried out following the instructions accompanying the GoTaq^®^ qPCR Master Mix kit from Promega. Each reaction solution consisted of the cDNA template, 2.5 microliters specific primers for each target gene, 5 microliters of double-distilled water, and 15 microliters of the qPCR Master Mix. Based on the NCBI database, the primer sequences employed in this study were carefully selected and are exhaustively detailed in **Table 1**. Relative transcript abundance of the target genes and the housekeeping gene were calculated according to 2−ΔΔCT formula.

**Table 1:** Primer sequences for gene expression in qRT-PCR analysis.

Genes	Gene ID	Primer sequence (5′→3′)	Length (bp)
*CpPCS*	111805552	ACTTCATCAGAAGCGGCCAA CCAAGCATAATGCACCACATCT	162
*CpPDH*	111809051	AATCTTGCTGTCTCTCCGGC ACGAGCCAACTCAACATCCA	186
*CpOAT*	111783359	TTCCTCCCCAAGTGATCCCA ATCCCACAGTGTGAGTCTACG	178
*CpARG*	111807154	TTCAACCCTCAGCGAGACAC ACCTTGGCTCATCACTCTCAC	187
*CpADC*	111794938	GGATAGCTCTCTTCCGTCGC CGAACGGTCATGTTCCCAGA	192
*CpODC*	111798957	CCAACATGGCCTCCTCCATT GGGTTGGGGTTGCATTTGAC	168
*CpGAD*	111808603	CCGTCGAAGGCCGATATCAA GCTCATCCGCTCTCACTTCA	176
*CpGAT*	111809776	CGTGGACTAAGGGTTGGAGTT ATGTGCGACAGACTGAGACC	200
*CpSSD*	111796065	CTTGGTCGGGAAGGATCCAA GGCCTCTGCCTATCTGGTTT	175
*CpAPX*	111782837	GCTAGTGGCATGGGGAGATG TCCAAAGAGACAGGGAGACCA	171
*CpMDR*	111809047	CGTTTGAGCAAGTGGGGTTG TGACACAATGCTGAGCCGAT	183
*CpACO*	111793728	AGGGAGCCTAACTTCCTCGT GCTCGAATCGTAGGTCCAGG	182
*CpAAO*	111789967	TGACGGCCTCAGAAAGTTCC CCGCCGACGATTATGACAGA	150
*CpECD*	111809781	ATGGATTCCGGCAGGAACAG CCCGCTGCAAGAAATTCCAC	169
*CpACTIN*	111787537	TCGGTGCCGAACGTTTTAGA AACCACCGGAGAGGACGATA	159

### 2.10 Statistical analysis

The present investigation was designed and performed following a thorough randomization method. Statistical analysis of the collected data was carried out using ANOVA model. Statistical comparisons among the treatments were conducted utilizing Duncan’s Multiple Range Test, with a significance threshold set at p-value less than 0.05.

## 3 Results

### 3.1 Chilling injury index, weight loss, MDA content and relative electrolyte leakage in zucchini fruit subjected to cold storage

**Figure 1:**
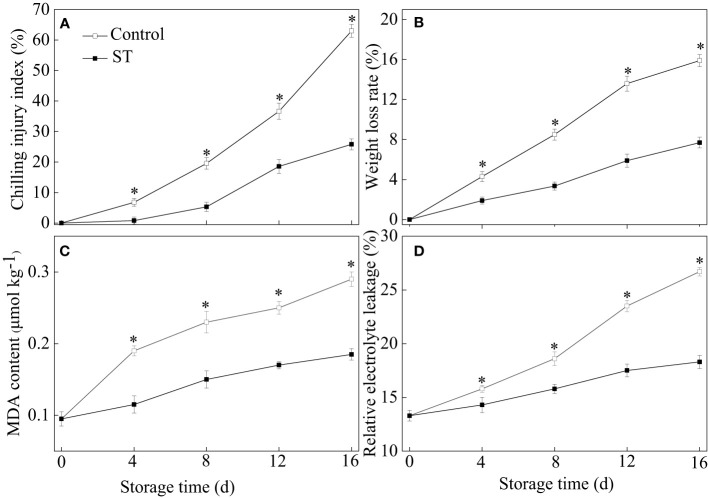
Variations of chilling injury index **(A)**, weight loss **(B)**, MDA content **(C)** and relative electrolyte leakage **(D)** in control and 5 μM ST treated zucchini fruit during cold storage. Vertical bars indicate the standard error of the mean values. Asterisks imply statistically significant differences (p < 0.05) between the control and ST treated group in the same day.

Throughout a 16-day period of cold storage at 4°C, a steady escalation in the chilling injury index was noticed in zucchini fruit. Zucchini fruit subjected to ST treatment exhibited a notably slower increase in the chilling injury index relative to untreated controls. After the 4th day of storage, the chilling injury index in ST-treated zucchinis was significantly less than in untreated ones (**Figure 1A**). The application of ST proved effective in curbing the surge of the chilling injury index. As the storage duration extended, there were gradual upsurges in weight loss rate, MDA content, and relative electrolyte leakage levels within zucchini fruit (**Figures 1B–D**). The findings from our experiments indicated that ST intervention significantly deterred the escalation of above three physiological parameters over time.

### 3.2 Proline content, metabolic enzymatic activities related to proline, and associated gene expression in zucchini fruit subjected to cold storage

**Figure 2:**
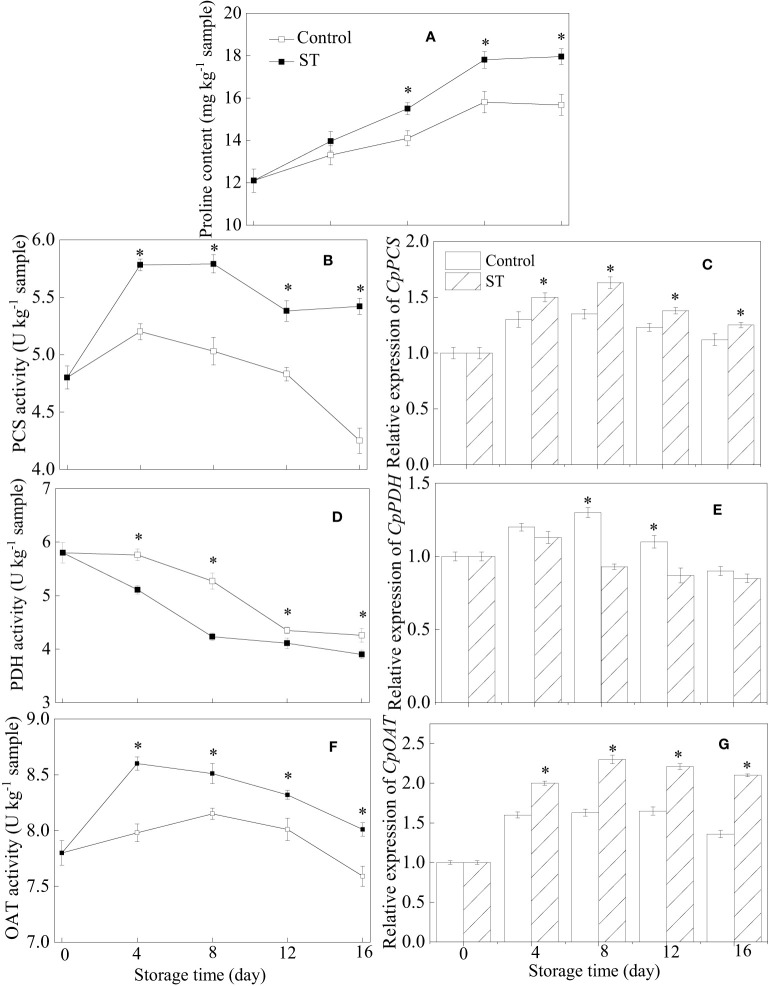
Variations of proline content **(A)**, metabolic enzymatic activities related to proline **(B, D, F)** and associated gene expression in control **(C, E, G)** and 5 mM ST treated zucchini fruit during cold storage. Vertical bars indicate the standard error of the mean values. Asterisks imply statistically significant differences (p < 0.05) between the control and ST treated group in the same day.

Throughout the entire storage period, proline content in zucchini fruit consistently rose (**Figure 2A**). Notably, this ascending trend was augmented in fruit that subjected to ST treatment. PCS activity in treated fruit initially displayed a pattern of rising before declining, reaching its peak at the eighth day of observation. Throughout the entire storage period, the control group consistently exhibited lower PCS activity levels compared to those in the ST-treated group (**Figure 2B**). Comparable patterns were also observed in the transcription abundance of *CpPCS* across both control and treated group (**Figure 2C**). PDH activity showcased a downward trend in zucchini fruit throughout the storage duration (**Figure 2D**). The expression of the *CpPDH* gene in zucchini fruit initially ascended before subsequently declining (**Figure 2E**). Remarkably, both the activity and transcription abundance of PDH were lower in the treated fruit compared to their counterparts in the control group. OAT activity in the ST-treated fruit registered a sharp rise during the initial four days of storage, followed by a gradual decrease over the rest of the storage timeframe (**Figure 2F**). Throughout the storage period, the expression of the *CpOAT* gene was consistently higher in the treated zucchini fruit compared to the control group (**Figure 2G**).

### 3.3 Arginine content, metabolic enzymatic activities related to arginine, and associated gene expression in zucchini fruit subjected to cold storage

**Figure 3:**
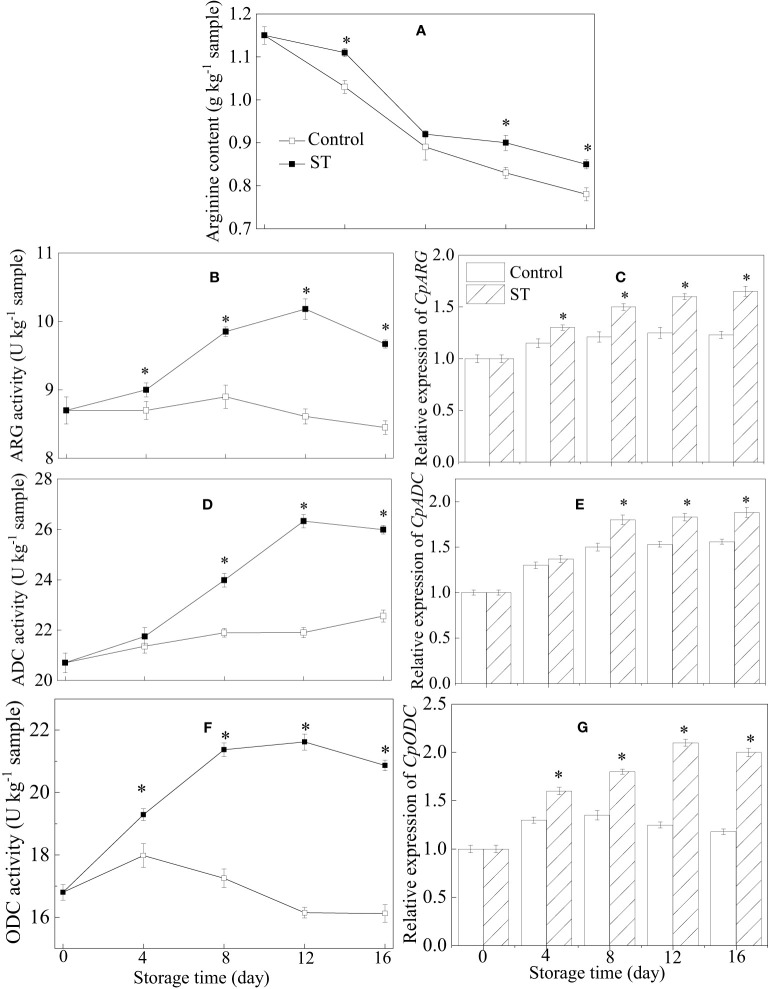
Variations of arginine content **(A)**, metabolic enzymatic activities related to arginine **(B, D, F)** and associated gene expression in control **(C, E, G)** and 5 mM ST treated zucchini fruit during cold storage. Vertical bars indicate the standard error of the mean values. Asterisks imply statistically significant differences (p < 0.05) between the control and ST treated group in the same day.

As can be observed in **Figure 3A**, a general decrease trend in arginine content is recorded during the course of storage duration (**Figure 3A**). Activities of ARG and ADC in treated fruit exhibited similar trajectories of ascent before descending, peaking at day 12. Over the entire storage period, the untreated fruit consistently presented with lower activities of ARG and ADC when compared to the treated counterparts (**Figures 3B, D**). ST treatment significantly increased the relative expression of *CpARG* and *CpADC* in zucchini fruit during cold storage (**Figures 3C, E**). Compared with the control group, ST treatment significantly promoted the increase of ODC activity and the relative expression of *CpODC* in zucchini fruit during cold storage (**Figures 3F, G**).

### 3.4 GABA content, metabolic enzymatic activities related to GABA, and associated gene expression in zucchini fruit subjected to cold storage

**Figure 4:**
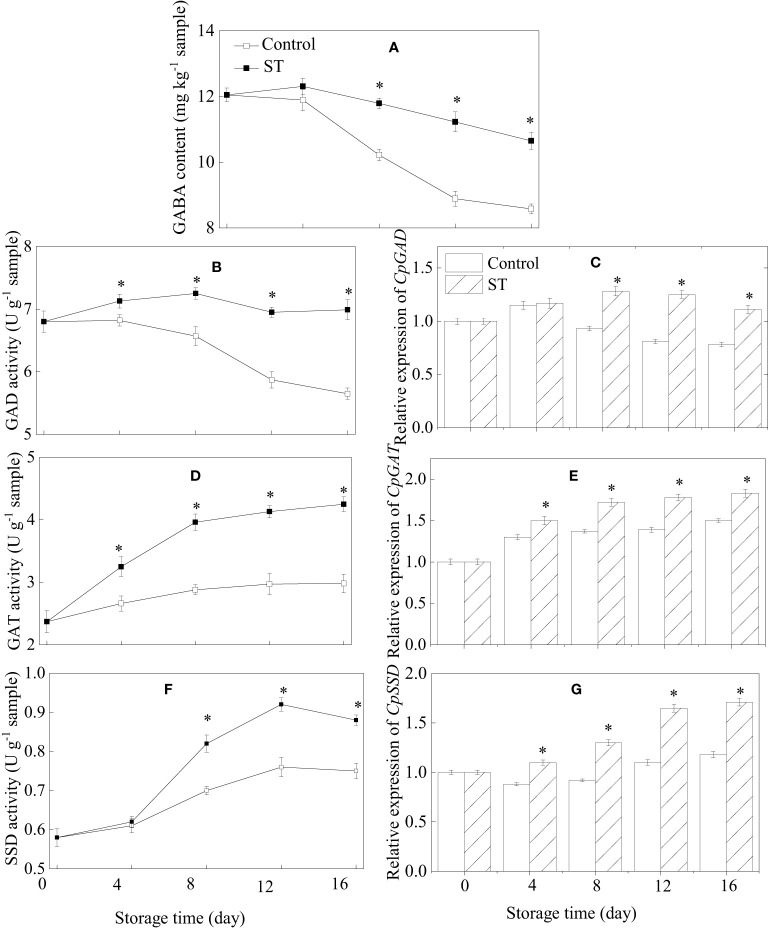
Variations of GABA content **(A)**, metabolic enzymatic activities related to GABA **(B, D, F)** and associated gene expression in control **(C, E, G)** and 5 mM ST treated zucchini fruit during cold storage. Vertical bars indicate the standard error of the mean values. Asterisks imply statistically significant differences (p < 0.05) between the control and ST treated group in the same day.

The content of GABA in control zucchini fruit consistently reduced over the course of their storage period. Conversely, in the ST-treated group, GABA levels peaked at day 4 and thereafter began to diminish (**Figure 4A**). GABA content in treated zucchini fruit was notably higher in comparison to the control fruit. The activity of GAD and expression of the *CpGAD* gene in zucchini fruit initially rose before declining later on. From the fourth day of storage onwards, both GAD activity and gene expression levels were significantly elevated in the treated fruit relative to the control fruit (**Figures 4B, C**). ST treatment resulted in a considerable enhancement of GAT activity and the expression of the *CpGAT* gene throughout the entire storage period (**Figures 4D, E**). The activity of SSD initially showed a significant increase followed by a slight diminution in both the control and treated group as storage duration extended (**Figure 4F**). In treated fruit, the relative expression of *CpSSD* consistently increased with prolonged storage, whereas in the control group, it initially decreased and then slightly rose as storage time lengthened (**Figure 4G**).

### 3.5 AsA content, metabolic enzymatic activities related to AsA, and associated gene expression in zucchini fruit subjected to cold storage

**Figure 5:**
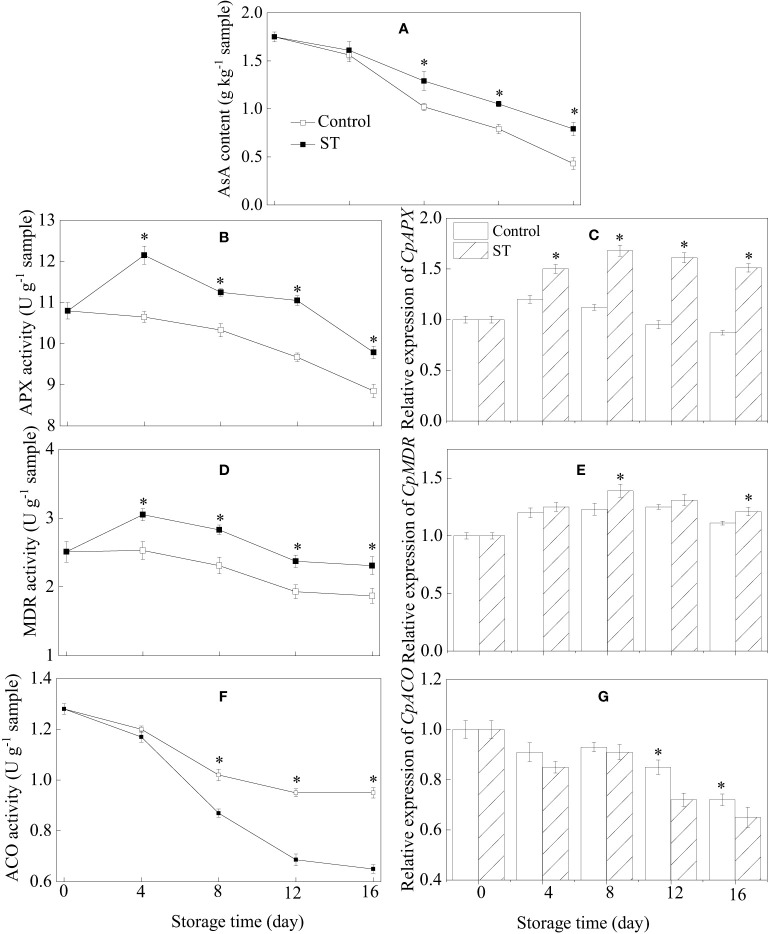
Variations of AsA content **(A)**, metabolic enzymatic activities related to AsA **(B, D, F)** and associated gene expression in control **(C, E, G)** and 5 mM ST treated zucchini fruit during cold storage. Vertical bars indicate the standard error of the mean values. Asterisks imply statistically significant differences (p < 0.05) between the control and ST treated group in the same day.

Over the course of storage, AsA content in zucchini fruit continuously diminished (**Figure 5A**). Nevertheless, the application of ST prior to storage served to alleviate this decline in AsA levels. In the ST-treated zucchini fruit, the activities of APX and MDR initially surged significantly before experiencing a modest decline as storage time progressed, consistently maintaining levels that were notably higher than those found in the control group (**Figures 5B, D**). Comparable trends in expression were also observed for the genes *CpAPX* and *CpMDR* in both control and treated zucchini fruit (**Figures 5C, E**). ACO activity in zucchini fruit exhibited a progressive decline throughout the entire storage period. Starting from the eighth day of storage, control samples displayed a notably higher ACO activity level compared to the ST-treated group (**Figure 5F**). The expression of *CpACO* gene in zucchini samples decreased over time (**Figure 5G**). However, after eight days of storage, the transcription level of *CpACO* was significantly downregulated in the ST-treated samples as opposed to the control fruit.

### 3.6 ABA content, metabolic enzymatic activities related to ABA, and associated gene expression in zucchini fruit subjected to cold storage

**Figure 6:**
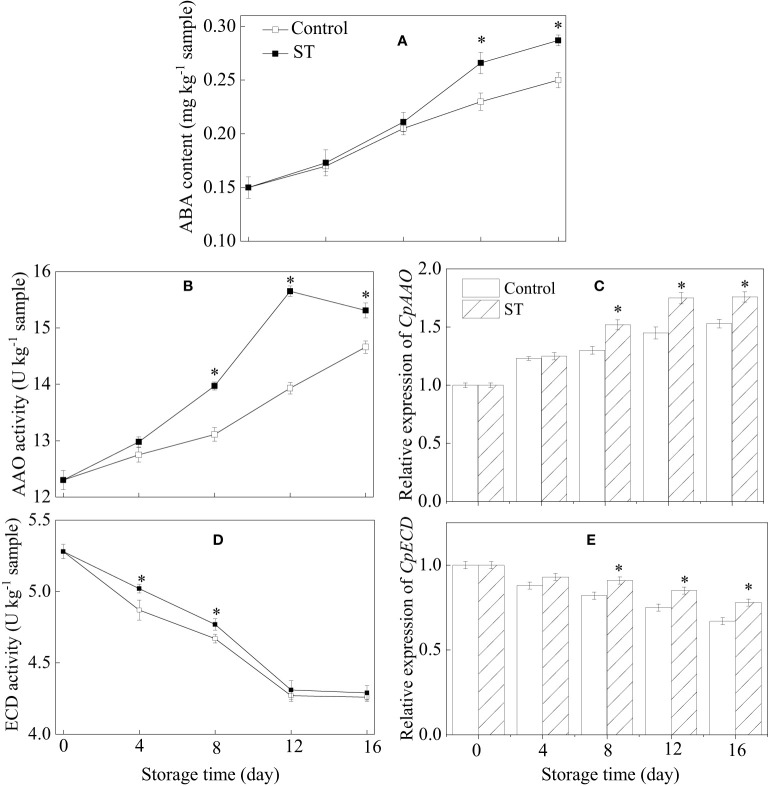
Variations of ABA content **(A)**, metabolic enzymatic activities related to ABA **(B, D)** and associated gene expression in control **(C, E)** and 5 mM ST treated zucchini fruit during cold storage. Vertical bars indicate the standard error of the mean values. Asterisks imply statistically significant differences (p < 0.05) between the control and ST treated group in the same day.

The results showed that ABA content rose continuously in both the ST-treated and control group, yet it was significantly higher in the ST-treated group specifically between the 12th and 16th days of the trial (**Figure 6A**). The activity of AAO in treated zucchini fruit initially rose before declining later on (**Figure 6B**). The expression of *Cp*AAO gene in zucchini samples increased gradually over time (**Figure 6C**). Starting from the eighth day of storage, ST treatment significantly heightened AAO activity and corresponding gene expression levels compared to the control fruit. During the complete storage span, activity of ECD and expression of *CpECD* gene in zucchini fruit consistently decreased over time. ST treatment induced a marked enhancement in ECD activity between the fourth and eighth days of the experiment (**Figure 6D**). In comparison to the control, the ST treatment notably augmented the increase in *CpECD* expression starting from the eighth day of storage (**Figure 6E**).

## 4 Discussion

Zucchini is a highly favored fruit enjoyed worldwide with perennial high consumer demand. Unfortunately, zucchini fruit displays an exceptional sensitivity to chilling injury when subjected to conventional cold storage methods. Chilling injury experienced by zucchini fruit during refrigeration is an irreversible physiological issue that significantly impacts its market worth and ability to resist diseases (Palma et al., 2019). Implementing effective postharvest strategies to prevent the progression of chilling injury is critically important for safeguarding the interests of the fruit sector. Growing body of robust evidence suggests that applying ST positively influences the activation of defense mechanisms and aids in mitigating various stress conditions (Marzec, 2016; Soliman et al., 2022). There is a rising interest in the metabolic pathways of internal signaling molecules and functional metabolites in relation to cold storage of horticultural commodities, given their integral participation in diverse physiological functions (Zhang et al., 2021). Earlier research has documented the favorable effects of ST treatment in diminishing chilling injury in several types of fruits, including litchi and pea (Cooper et al., 2018; Liu et al., 2024a). In our current investigation, we observed that immersing zucchini fruit in a solution containing 5 μM ST effectively reduced the occurrence of chilling injury during refrigeration.

It is widely accepted that the development of chilling injury in fruit and vegetable largely stems from cellular membrane damage occurring during the postharvest storage phase (Zhang et al., 2021). The cell membrane constitutes the principal location where chilling injury takes root. When cell membranes undergo a transition from their fluid, liquid-crystalline state to a rigid, solid-gel structure under chilling temperatures, it significantly raises the likelihood of compromised regulated semi-permeability, leading to potential cell membrane dysfunction (Liu et al., 2016). The incidence of chilling injury is frequently linked with oxidative damage, which can be biochemically evidenced by the presence of MDA—a key marker representing the endpoint of lipid peroxidation processes. Both electrolyte leakage and MDA accumulation are well-regarded proxies for assessing the extent of damage to semipermeable membranes, typically caused by oxidative degradation of membrane lipids. These parameters are frequently utilized in determining the functional integrity of the cell membrane (Wang et al., 2023). Increased relative electrolyte leakage in plants promotes more efficient interactions between enzymes and substrates, thereby accelerating intracellular oxidation reactions. Malondialdehyde content serves as an indicator of the severity of oxidative stress endured by the plant (Mohammadi et al., 2023). A wealth of research literature decisively suggested that a reduction in relative electrolyte leakage and MDA accumulation could significantly bolster the chilling tolerance of a variety of horticultural commodities. Zhang et al. (2023) substantiated that a decrease in relative electrolyte leakage and MDA levels jointly contributed to alleviating chilling injury in cucumber fruit when exposed to fucoidan. Our findings showed a consistent rise in electrolyte leakage and MDA levels across both control and treated zucchini fruit. Nonetheless, the application of ST significantly postponed this escalation, consequently attenuating the progression of chilling injury in the treated zucchini fruit. It can be deduced that the application of ST likely contributes to enhancing chilling resistance in zucchini fruits by virtue of its ability to reduce electrolyte leakage and MDA levels.

Within plant organisms, proline operates as a signaling agent that engages in numerous biochemical reactions, particularly during adverse environmental stress scenarios. Not only does proline serve as an effective osmoprotectant through its compatibility as a solute, but there is compelling evidence suggesting that it also plays crucial roles in neutralizing free radicals, regulating cellular redox status, sequestering metal ions, and instigating protective responses within plants (Raza et al., 2023). Proline is widely recognized for its capacity to augment plant resilience against chilling stress. Ample research supports the involvement of multiple enzymes in proline metabolism, which is crucial for sustaining a fundamental equilibrium in plants. The formation of proline from either glutamate or ornithine is catalyzed by different enzymes; PCS operates in the cytosol while OAT functions in plastids. In parallel, proline breakdown takes place within mitochondria, involving several interconnected metabolic steps driven by a series of enzymes, such as PDH among others (Mattioli et al., 2009). A pronounced accumulation of proline represents a significant adaptive physiological trait contributing to plant resilience against various stress conditions. Notably, in plants susceptible to cold stress, a heightened proline buildup can alleviate the detrimental effects of chilling injury (Wang et al., 2023). There is a mounting body of evidence indicating that the modulation of enzyme activities and gene expression, which in turn affects proline levels, can help mitigate CI in harvested horticultural products during postharvest storage. Aghdam et al. (2019) discovered that melatonin treatment conferred chilling resistance to tomato fruit through the regulation of enzymes associated with proline metabolism, ultimately impacting proline levels. Islam et al. (2022) also reached comparable conclusions, suggesting that the augmented proline levels resulting from heightened PCS and OAT activities and suppressed PDH activity were instrumental in boosting chilling tolerance in pomegranate subjected to 24-epibrassinolide treatment. Significantly, the upregulated expression of genes related to proline metabolism has been shown to confer greater resilience to environmental stressors. This correlation extends to the response of pear fruit to chilling injury. Sun et al. (2020) discovered that glycine betaine induced chilling tolerance in pear fruit was linked with heightened transcriptional levels of *PuPCS* and *PuOAT*, alongside reduced expression of *PuPDH*, all of which are genes connected to proline metabolism. Increasing proline level during cold storage within plants is a common phenomenon and arises as a net result of augmented biosynthesis and altered degradation processes. The elevated proline levels observed in zucchini fruit during cold storage can be attributed to its innate defense mechanism against cold stress, which stimulates the metabolic pathway of proline synthesis. The observed enhancement seems to stem from the zucchini fruit’s reaction to cold stress, compounded by the influence of ST treatment, which further induces proline metabolism. Thus, it is reasonable to infer that ST treatment’s positive impact on elevating enzyme activities and gene expressions leads to increased proline accumulation, which in turn aids in reducing the severity of chilling injury.

Arginine, a fundamental building block of proteins and a source for generating certain bioactive substances in higher plants, is a pivotal amino acid deeply integrated into various metabolic processes and actively engaged in the stress response mechanisms of these plants (Nasr et al., 2022). As arginine possesses a high carbon-to-nitrogen ratio, it not only supplies plants with essential nitrogen but also sustains cellular functions and boosts tolerance to diverse abiotic stresses. Its metabolism provides a platform for several ornithine-dependent pathways, such as the production of polyamines and proline, which contribute to stress adaptation (Alcázar et al., 2010). Arginine has drawn widespread interest in research due to its proven effectiveness in improving cold tolerance in harvested crops during postharvest storage. A variety of enzymes participate in the breakdown of arginine, such as arginase and arginine decarboxylase (ADC). ADC enzymatically converts arginine into polyamines; meanwhile, arginase breaks down arginine into urea and ornithine. Ornithine serves as a precursor for both polyamines and proline production through two separate pathways—ornithine decarboxylase (ODC) generates polyamines, while ornithine aminotransferase contributes to proline biosynthesis (Zhang et al., 2013). Additionally, an elevation in arginine content and augmented arginase activity could play a part in ensuring regular cell operations and preserving structural stability (Bokhary et al., 2020). Enhancing chilling tolerance in zucchinis upon application of hot water treatment may be attributed to the increased accumulation of endogenous arginine, facilitated by heightened expression of ADC and ODC genes and corresponding enzyme activities (Bokhary et al., 2020). Similar findings were also seen in papaya fruit treated with arginine, indicating that the acquired chilling tolerance was a result of the synergistic regulation of ARG, ODC, and ADC enzyme activities (Huang et al., 2021b). In our research, we found that the application of ST treatment to zucchini fruit under cold storage conditions led to augmented activities of ARG, ODC, and ADC enzymes, accompanied by a corresponding increase in the expression of their respective genes. Moreover, the elevated enzyme activities and corresponding gene expressions in ST-treated zucchini fruit resulted in higher arginine content, ultimately contributing to enhanced chilling tolerance during refrigerated storage. Based on the aforementioned findings, we could deduce that compared to control fruit, the augmented activities and gene expressions of ARG, ADC, and ODC enzymes in ST-treated zucchini lead to a higher arginine content, which may significantly contribute to the improved chilling tolerance regulated by ST in harvested zucchini fruit.

Several research studies have convincingly demonstrated the complex and diverse roles that endocellular GABA plays in plant growth and development, acting as a signaling agent that influences a wide array of physiological processes. These functions encompass stress responses to growth control, extending to challenges posed by both living organisms and environmental factors (Kinnersley and Turano, 2010; Bown and Shelp, 2016). GABA is also recognized as a stress-responsive metabolite and maintains a strong connection with other intrinsic phytohormones (Palma et al., 2019). In the context of plants, GABA is produced through a specialized metabolic route known as the GABA shunt pathway, a process meticulously managed by a sequence of enzymes—chief among them being SSD, GAT, and GAD (Tarkowski et al., 2020). The enzyme GAD plays a core role in GABA metabolism by facilitating the enzymatic reaction that converts glutamate into GABA. Subsequently, GABA experiences breakdown via the process of transamination. On the other hand, GAT enzyme plays a crucial role in reversibly transforming GABA into succinic semialdehyde via deamination. The ensuing succinic semialdehyde is then converted into succinic acid by SSD. Finally, succinic acid gets processed within the TCA cycle (Tarkowski et al., 2020). The fluctuations in GABA concentration are intricately linked with adverse conditions, such as chilling injury, in a wide array of horticultural species. An increase in GABA levels during cold storage represents a natural physiological adaptation, emerging from the delicate equilibrium between its production and breakdown processes. Multiple researches have convincingly revealed that augmenting chilling tolerance in harvested produce involves stimulation of enzyme activities and the activation of genes encoding for elements of the GABA shunt pathway. In the case of Pyrus ussuriensis, treating the fruit postharvest with CaCl_2_ has been proven to enhance its resistance to chilling stress by influencing the enzymes and genes tied to GABA metabolic processes (Li et al., 2020). Analogously, it was discovered that loquats treated with phytosulfokine α (PSKα) showed augmented activities of GAD and GAT, which were instrumental in combating postharvest chilling stress (Liu et al., 2024c). In present investigation, we observed that ST treatment in zucchini fruit undergoing cold storage significantly boosted the activity levels of the enzymes of SSD, GAT, and GAD, concurrent with an increase in the expression of the corresponding genes. This upregulation corresponded to a heightened GABA content in the ST-treated zucchini fruit, which was pivotal in fostering enhanced chilling tolerance during storage under low temperature. The findings indicated that ST treatment led to an increased accumulation of GABA in zucchinis potentially by stimulating the activities and gene expressions of GAD, GAT, and SSD, effectively mitigating chilling stress impacts.

Ascorbic acid, commonly referred to as vitamin C, is an essential antioxidant abundantly present in plant life. It significantly influences fruit ripening processes and stress resilience, and exerts a crucial regulatory function in fruit maturation and preservation during postharvest storage (Smirnoff, 2018). Increasing levels of AsA within fruit not only enrich the trophic profile of the fruit but also bolster its resistance against a broad spectrum of environmental stress factors. The AsA content in fruit tissues is influenced not just by its synthesis but also by its degradation and regeneration, where the ascorbate–glutathione cycle can potentially stimulate AsA accumulation (Liu et al., 2024b). An accumulating body of evidence indicates that a suite of recycling enzymes play a significant role in the AsA metabolic pathway. Utilizing ascorbate as a reactant, the enzyme APX effectively neutralizes excessive hydrogen peroxide, thus guarding cells against oxidative stress damage. Additionally, APX orchestrates the chemical transition of AsA into monodehydroascorbic acid. Simultaneously, monodehydroascorbic acid can revert back to AsA through the enzymatic action of MDR. Ascorbate oxidase, an enzyme of the ascorbate oxidase family, also contributes to the conversion of a portion of AsA into monodehydroascorbic acid through its catalytic action (Luo et al., 2022). Experimental data has convincingly shown a profound interconnection between the incidence of chilling injury and the metabolic processes involving AsA in postharvest horticultural products. According to our previous investigation, the application of melatonin on harvested *Pyrus bretschneideri* fruit led to an improvement in their cold resistance. This enhancement was linked to the increased enzyme activities and gene expression of APX and MDR, which in turn resulted in a rise in ascorbic acid (AsA) content (Liu et al., 2024b). There is evidence suggesting that, in comparison with the biosynthesis of AsA, the cyclic degradation pathways play a potentially more significant role in sustaining the AsA levels within postharvest fruit (Luo et al., 2022; Zheng et al., 2022). For example, Luo et al. (2022) disclosed that subjecting kiwifruit to melatonin treatment led to a decrease in the expression of *AcACO* and increase in the transcriptional level of *AcAPX* and *AcMDR* related to AsA metabolism during cold storage, consequently postponing the onset of chilling injury. In this study, ST treatment was found to enhance the chilling tolerance of zucchini fruit, a process that coincided with heightened activities and gene expressions of APX and MDR enzymes, reduced ACO activity and gene expression, and elevated AsA concentration. Based on these findings, it is hypothesized that the AsA metabolism triggered by ST treatment plays a critical role in inducing improved chilling tolerance in zucchini fruit stored under cold conditions.

Abscisic acid (ABA), a key plant hormone, exerts a vital influence over multiple stages of plant growth and development. Serving as a central regulatory element, ABA orchestrates plants’ resistance and adaptive responses to a variety of environmental stressors, particularly those that lead to dehydration, including drought, high salinity, and low temperatures (Chen et al., 2020). The synthesis of ABA progresses through a chain of precisely orchestrated enzymatic steps. The enzyme ECD facilitates the oxidative breakage of 9’-cis-neoxanthin and 9’-cis-violaxanthin, giving rise to xanthoxin. Next, this precursor molecule is converted into abscisic aldehyde via the catalytic action of a short-chain alcohol dehydrogenase. Ultimately, AAO enzyme performs the oxidative reaction that transforms abscisic aldehyde into the final product, ABA (Wu et al., 2023). ECD stands out among these enzymes as a pivotal regulatory component that significantly influences the pace of ABA biosynthesis, acting as a pivotal rate-controlling enzyme in the process. Research indicates that there is a positive correlation between an increase in the internal production of ABA and a decrease in the severity of chilling damage experienced by litchis and zucchinis (Zuo et al., 2021; Liu et al., 2024a). Carvajal et al. (2017) highlighted that during cold storage, zucchini fruit exhibited an upsurge in the inherent abscisic acid levels as well as an enhancement in the activity of AAO, an enzyme integral to ABA biosynthesis. The experimental observations strongly suggest that ABA serves as the key phytohormone contributing to the enhancement of cold tolerance in harvested zucchini fruit during postharvest storage (Carvajal et al., 2017; Zuo et al., 2021). On the contrary, the results presented by Xu et al. (2019) have established that UV-C treatment efficiently postpones the senescence of strawberry fruit by downregulating the expression of genes related to ABA biosynthesis, thereby reducing ABA levels. This variation can be explained by considering the distinct attributes of individual fruit cultivars along with the inherent peculiarities of the diverse fruit tissues. Our research revealed that soaking zucchini fruit in ST solution led to an elevation in ABA concentration, concurrent with increases in both the activities and transcriptional abundance of AAO and ECD enzymes. On this basis, we conjecture that the abscisic acid metabolic alterations induced by ST application contribute to alleviating chilling injury in zucchini fruit during refrigerated storage.

## 5 Conclusions

In summary, our experimental findings validated that applying ST after harvest proved to be effective in diminishing the effects of chilling injury in zucchini fruit during storage at the low temperature of 4°C. ST treatment led to a decline in the rate of weight loss and a reduction in both relative electrolyte leakage and MDA content in zucchini fruit stored under cold condition for a duration of 16 days. ST treatment triggered an elevation in the levels of proline, arginine, AsA, GABA, and ABA, which was accomplished by means of modulating the enzymatic activities and transcriptional levels of corresponding genes within the zucchini fruit. The foregoing findings suggest that the postharvest application of ST presents a viable strategy to alleviate CI in zucchini fruit when stored under low temperature condition.

## Data availability statement

The original contributions presented in the study are included in the article/**Supplementary Material**. Further inquiries can be directed to the corresponding author.

## Author contributions

LW: Data curation, Formal analysis, Investigation, Validation, Writing – original draft. LL: Data curation, Formal analysis, Methodology, Writing – original draft. AH: Formal analysis, Investigation, Methodology, Writing – review & editing. HZ: Formal analysis, Methodology, Project administration, Writing – review & editing. YZ: Conceptualization, Supervision, Writing – review & editing.
