# Supplementary material for: The metabolism of amino acids, AsA and abscisic acid induced by strigolactone participates in chilling tolerance in postharvest zucchini fruit
Source: Front Plant Sci. 2024 May 14;15:1402521. doi: 10.3389/fpls.2024.1402521 (PMC11130489; doi:10.3389/fpls.2024.1402521)
Supplement: Supplementary file 1 [file DataSheet_1.docx]

In the preliminary screening experiment, we treated zucchini fruit with 0 μM, 1 μM, 5μM, 10 μM, and 20 μM of ST, and certified that the concentration of 5 μM had optimal effectiveness. The figure below is the chilling injury index in zucchini fruit under different concentrations of ST during cold storage.
